# RetinoMamba-AnatomyQuery: Anatomy-Guided Query Learning for Efficient Vision Impairment Screening from Retinal Fundus Images

**DOI:** 10.3390/life16091577

**Published:** 2026-09-21

**Authors:** Abdul Rahaman Wahab Sait, Yazeed Alkhurayyif

**Affiliations:** 1 Department of Documents and Archive, Center of Documents and Administrative Communication, King Faisal University, Al-Ahsa 31982, Saudi Arabia; 2 Applied College, Shaqra University, Shaqra 11961, Saudi Arabia

**Keywords:** vision impairment screening, retinal fundus imaging, vision mamba, anatomy query learning, retinal disease classification, explainable artificial intelligence

## Abstract

Automated analysis of fundus images is a promising technique for efficient, scalable retinal screening. In real-time screening, binary classification provides a straightforward first-line solution by differentiating normal and abnormal images, enabling prioritization of potentially abnormal cases for subsequent medical and disease-specific analysis. This study proposes RetinoMamba-AnatomyQuery, a lightweight model that integrates Vision Mamba-based global contextual representations, EfficientNet-B3-derived local features, anatomy–lesion query refinement, and anatomy-aware feature fusion. The Ocular Disease Intelligent Recognition (ODIR)-2019 dataset and Brazilian Multilabel Ophthalmological Dataset (BRSET) are harmonized to construct the internal cohort, yielding 10,842 eligible unique patients following expert-assisted quality control and data curation. The proposed model achieves accuracy of 97.93%, recall of 97.77%, specificity of 98.07%, precision of 97.87%, and an F1-score of 97.82%, outperforming state-of-the-art models. Independent evaluation on the external dataset (Retinal Fundus Multi-disease Image Dataset (RFMiD)) reports an accuracy of 96.21% and an F1-score of 95.99%, demonstrating sustained performance across an independent data distribution. Computational evaluation demonstrates a controlled footprint of 23.9 million parameters and 8.3 giga floating-point operations, supporting computationally efficient screening. Overall, the findings demonstrate the effectiveness of anatomy-conditioned global–local feature integration for reliable normal/abnormal fundus discrimination.

## 1. Introduction

The problem of visual impairment and blindness continues to be one of the most prevalent global public health challenges, affecting millions of individuals and causing a significant social and economic burden on healthcare systems across the world [1,2,3]. Retinal diseases, including diabetic retinopathy, age-related macular degeneration, glaucoma, and retinal vasculopathy, cause a large proportion of visual impairment [4]. The key feature of most of these diseases is gradual degradation of retinal tissue. Thus, timely screening plays a critical role in avoiding irreversible impairment and blindness. Early diagnosis of abnormal retinal findings enables treatment of underlying diseases and helps prevent negative outcomes [5]. Therefore, retinal screening has become an integral part of ophthalmology and vision care.

Retinal fundus photography is one of the most popular approaches to visualizing the retina because it is cost-effective, simple, and provides a comprehensive overview of retinal anatomy and pathology [6,7,8]. Using these images, ophthalmologists can assess the optic disc, macula, fovea, and retinal vessels to identify microaneurysms, hemorrhages, exudates, and vessel lesions [9,10,11]. Anatomy-centered examination plays a crucial role in determining disease severity and risk, as well as identifying individuals who require additional expert examination [12,13].

Distinguishing between normal and abnormal retinal images is essential for further diagnosis and disease grading [14]. Thus, binary classification of retinal images is an important clinical task that serves as a preliminary step prior to disease diagnosis. From a healthcare perspective, highly accurate normal/abnormal detection systems can significantly reduce the burden on specialists by focusing on abnormal images and filtering out normal ones [15]. Additionally, as abnormal retinal images encompass a wide range of diseases with unique visual symptoms, binary screening requires developing generalized representations of retinal health rather than disease-specific patterns [16].

Recent advances in artificial intelligence have greatly improved automated screening systems in accuracy, efficiency, and reliability [16]. Specifically, deep learning models based on convolutional neural networks (CNNs) have shown impressive ability to learn hierarchies of features in retinal images and have delivered promising results in ophthalmology. Researchers have used ResNet (ResNetV2, 2016), DenseNet (DenseNet-BC, 2017), EfficientNet (EfficientNetV2, 2021), and other variations to classify images, detect lesions, and predict disease risk [17]. However, current CNN-based architectures rely heavily on local convolutions and can struggle to capture relationships between distant regions of the retina [17]. Because anatomical structures interact in complex ways, efficient screening requires understanding both local and global retinal context.

Therefore, transformer architectures have become increasingly popular in medical image analysis. Vision transformers (ViTs), with their self-attention mechanism, have shown promising results for medical image classification, segmentation, and generation [18]. Although these algorithms have achieved remarkable performance in retinal image classification, transformers have quadratic computational costs with respect to input resolution, making them unsuitable for processing high-resolution fundus images [19]. Moreover, standard self-attention is purely data-driven and lacks intrinsic alignment with clinically meaningful anatomical structures [20]. Thus, attention may select locations that are not always clinically meaningful, limiting prediction interpretability.

In contrast to transformer models, state-space models (SSMs) and Vision Mamba provide opportunities for efficient visual representation learning [21]. Vision Mamba combines selective state-space modeling with localized feature extraction to enable effective long-range dependency learning without excessive complexity [22]. Therefore, Vision Mamba is particularly well suited for retinal image analysis, which requires capturing both local lesions and global structures. Although it models long-range context dependencies effectively at significantly lower computational cost than conventional transformer-based models, Vision Mamba relies primarily on data-driven feature learning without incorporating anatomical expertise [23]. Thus, its performance is similar to existing retinal image analysis pipelines, as it learns optimal feature maps solely by optimizing classification accuracy, without considering anatomical relationships and their effects on pathology. This approach fails to extract anatomically driven features and therefore limits interpretability and model transparency in automated clinical decision-making.

Retinal abnormalities are identified by anatomical location rather than visual examination alone [24]. Lesion significance is determined by its proximity to key retinal structures such as the optic disc, macula, fovea, and vasculature [25,26]. For instance, lesions in the macular area usually cause central vision problems, and changes around the optic disc are associated with glaucoma. Although anatomical context is indispensable for clinical diagnosis of retinal diseases, current deep learning methods for retinal image classification rarely account for it.

Existing approaches aim to incorporate anatomical knowledge into deep learning through segmentation-based learning, landmark localization, attention mechanisms, and graph-based modeling of relationships among anatomical regions. All these models produce remarkable results; however, they usually involve either graph structure construction or attentional operations, which add model complexity, increase training time, and depend heavily on segmentation or landmark detection accuracy. These factors affect model performance and applicability in large-scale screening. This study aims to develop a computationally efficient, anatomically interpretable framework for automated retinal screening that accurately distinguishes normal retinal images from abnormal cases associated with vision-threatening conditions. To achieve this objective, the proposed framework combines global contextual modeling, local lesion representation, anatomy-aware query learning, and auxiliary anatomical supervision within a unified end-to-end architecture. This study introduces RetinoMamba-AnatomyQuery, an anatomy-guided retinal screening framework that integrates Vision Mamba’s efficient global representation with an inherent anatomy query module. The contributions of this study are summarized as follows:

1. An inherent anatomy query module is proposed to encode anatomical relationships through anatomically initialized learnable queries, eliminating the need for explicit graph construction while enabling anatomy-aware representation learning.
2. A lightweight anatomy-aware feature fusion strategy with auxiliary segmentation supervision is introduced to integrate global contextual and anatomical representations within a unified learning framework.
3. An anatomy-guided Vision Mamba framework is developed for binary retinal screening by jointly learning global contextual, local lesion, and anatomy-aware feature representations.

Unlike existing studies, the proposed method learns a compact set of anatomically initialized queries within a deformable attention framework, thereby enabling more efficient interaction between the query-based anatomical representation and multi-scale retinal feature maps. In addition, the proposed feature fusion strategy integrates anatomy-driven feature representation with the global context through a Feature-wise Linear Modulation (FiLM) block, yielding a discriminative representation for binary retinal screening, similar to the anatomy-based diagnostics performed by clinicians.

The remainder of this study is structured as follows. Section 2 reviews recent developments in artificial intelligence-based retinal image analysis, Vision Mamba, and anatomical knowledge-based learning, highlighting gaps in the literature to explain the rationale for the proposed approach. Section 3 describes the proposed methodology, covering data pre-processing, network architecture, the anatomy query module, and the optimization procedure. Section 4 presents the experimental evaluation and comparative analysis. Section 5 discusses the results, the clinical relevance of this study, its limitations, and avenues for future research. Finally, Section 6 concludes the study by summarizing the principal findings and their implications for automated retinal screening and early detection of vision impairment.

## 2. Literature Review

Recent advances in artificial intelligence have greatly improved the ability to perform automated classification of retinal diseases, primarily through large, publicly available fundus image datasets and established algorithm testing methods. Public datasets such as EyePACS [27], APTOS [28], ODIR-2019 [29] BRSET [30,31,32,33], and RFMiD [34,35] have greatly assisted in developing deep learning models for retinal classification, including detection of diabetic retinopathy, glaucoma, age-related macular degeneration, and multi-disease classification. As a result of recent developments, researchers are increasingly shifting their focus from detecting the presence of one disease to designing a system that can be used for generalized retinal screening and large-scale ophthalmology screening programs.

Despite this progress, most studies focus on multi-class disease recognition or classification into several progression levels, while few studies address binary classification tasks that identify normal/abnormal stages [36]. Binary screening is the initial step in an ophthalmologist’s workflow and serves as the triage method for directing patients who need further specialist evaluation [37]. Several recent studies pointed out that generalized screening systems should be characterized by high sensitivity, the ability to recognize various disease manifestations, and adequate performance with images acquired under varied lighting conditions [38]. Despite the growing number and diversity of image databases, inter-dataset variability, lesion heterogeneity, class imbalance, and imaging protocols remain challenges for improving the generalization of existing models.

To increase the clinical significance of retinal image analysis using deep learning, researchers incorporate more anatomical information into deep learning models [39]. Unlike traditional methods that rely only on lesion characteristics, recent approaches using CNNs and ViTs incorporate features such as the optic disc, macula, fovea, and blood vessel network to extract crucial information [40,41,42,43,44,45]. Goh et al. [43] compared multiple CNNs and ViTs for binary referable diabetic retinopathy detection. Hwang et al. [44] and Alayon et al. [45] demonstrate the role of CNNs and ViTs in identifying glaucoma using fundus images. They outline the influence of training distribution, image quality, and domain shift. Likewise, Abramovich et al. [46], Yang et al. [47], Chaurasia et al. [48], and Saproo et al. [49] highlight the discriminative power of CNNs and ViT architectures. In addition, ATLASS [50] leveraged vessels and optic disc information in self-supervised pre-training through an anatomically guided masking scheme. While the field has advanced, researchers have mostly used anatomical information as an auxiliary supervision source.

Recently, learnable queries have been suggested as an alternative framework for structured feature representation in computer vision problems [51]. Instead of dense feature interactions and self-attention mechanisms, learnable query representations retrieve crucial information using limited embeddings. Consequently, learnable queries have shown successful results in image segmentation. With the introduction of the Segment Anything Model (SAM) [52,53] and its derivative, MedSAM, prompt and query embeddings have provided an efficient way to locate anatomic structures across different medical image formats.

Current studies have applied Vision Mamba [54] with feature extractors to improve diabetic retinopathy grading and lesion localization algorithms. Researchers have used Vision Mamba in models with enhanced attention and multilevel architectures to capture retinal anatomical features at different scales. It has been used with networks for retinal vessel segmentation and multimodal diagnostic tools for retinal disease classification [55]. However, most existing approaches use the Vision Mamba architecture as a feature-extraction backbone without incorporating anatomical information into feature learning [56]. In addition, the interaction between the anatomical prior and the state-space visual features still needs further investigation.

Foundation models are increasingly important for medical image classification, as large-scale pre-training enables them to learn transferable visual representations from labeled and unlabelled data [57]. These representations reduce dependence on extensive task-specific annotations and support effective adaptation to smaller clinical datasets. In fundus image analysis, foundation models can capture local lesions and global retinal structures while improving robustness to variations in cameras, populations, and acquisition conditions. Retinal-specific models, including RETFound, and self-supervised ViT-based approaches, including GONet, demonstrate their value for disease screening [58]. Nevertheless, external validation, bias assessment, interpretability, and computational efficiency remain essential for reliable clinical deployment and equitable diagnostic performance.

Overall, recent studies have made significant progress in retinal image analysis through developments in anatomy-aware learning, query-driven representation learning, and Vision Mamba-based architectures. However, these directions have developed independently. Anatomy-guided retinal image analysis approaches use structural information as auxiliary supervision. Vision Mamba-based approaches also mostly use SSM models as feature-extraction backbones without incorporating explicit anatomical prior knowledge. Moreover, learnable queries effectively produce structured feature representations but remain underexplored in retinal classification. These observations indicate a clear need for a unified framework that leverages anatomical information, state-space visual representations, and query-based representation learning for retinal classification.

## 3. Materials and Methods

**Figure 1:**
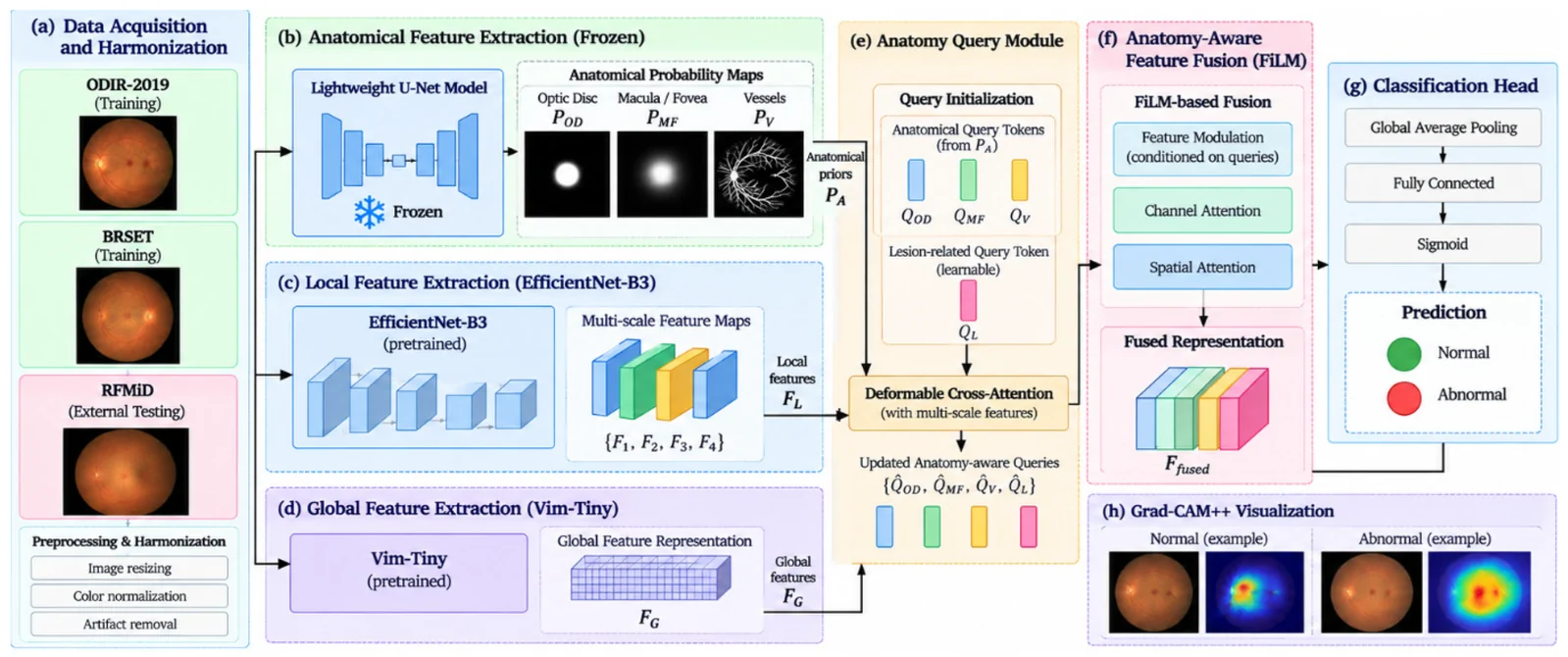
The proposed research methodology. (**a**) Dataset acquisition, preprocessing, and harmonization, (**b**) Frozen anatomical-prior extraction, (**c**) EfficientNet-B3 local feature extraction, (**d**) Vim-Tiny global feature extraction, (**e**) Anatomy-guided query initialization and refinement, (**f**) Anatomy-aware FiLM feature fusion, (**g**) Binary Normal/Abnormal classification, and (**h**) Grad-CAM++ visualization of model predictions.

The proposed methodology addresses complementary requirements for global contextual modeling, local pathology characterization, anatomical understanding, and explainability in retinal screening, as shown in Figure 1. Vision Mamba models long-range retinal dependencies with linear computational complexity. In contrast, the CNN branch retains fine-grained local abnormalities, which can become less prominent during global feature modeling. To introduce structural context, the system uses anatomical and lesion segmentations as spatial priors for the anatomy query module, where anatomically initialized and lesion-directed queries select relevant information from multi-scale representations via deformable attention. The query-based approach eliminates the need to construct an explicit graph while retaining interactions between anatomy and pathologies in the learned representation. FiLM combines information from anatomical segmentation and global features to perform binary classification. Lastly, Grad-CAM++ provides post hoc localization of the relevant image regions for each model prediction.

### 3.1. Dataset Acquisition and Preparation

Three publicly available color fundus image datasets, including ODIR-2019, BRSET, and RFMiD, were utilized to construct training and external validation datasets for model development. The ODIR-2019 dataset [29] consists of 10,000 fundus images belonging to 5000 patients, while the BRSET dataset [30,31,32,33] contains 16,266 fundus images from 8524 patients. The datasets were used for developing the model with patient-level splitting using a patient identifier, where all the images pertaining to a certain patient belonged to the same dataset split in order to avoid the leakage of subject-level information. The original diagnostic labels in datasets were transformed into a single binary label: images with no pathologies were labeled normal, and those with ocular pathologies were labeled abnormal. RFMiD [34,35] contains 3200 fundus images and was retained exclusively as an independent external test cohort, providing cross-dataset assessment without contributing to model training or parameter selection.

### 3.2. Dataset Harmonization and Cohort Curation

After harmonizing patient identifiers and integrating datasets, duplicate records and samples with unresolved patient identifiers, incomplete diagnostic information, ambiguous class assignments, or insufficient image quality were excluded. Clinical ophthalmology experts reviewed diagnostically uncertain cases and verified the clinical consistency of the normal/abnormal categorization during quality control and label harmonization. Images for which diagnostic interpretation or class assignment could not be established with sufficient confidence following expert assessment were excluded from subsequent analysis. This structured curation process reduced the initial combined cohort to 10,842 eligible unique patients, which constituted the final internal dataset. Patient-level partitioning was subsequently applied to five-fold cross-validation, ensuring that all fundus images associated with an individual patient remained exclusively within a single fold and preventing patient-level information leakage [59,60].

For independent external validation, 20% of the RFMiD dataset was selected using stratified random sampling with a fixed random seed, preserving the normal/abnormal class distribution of the complete dataset. The 20% subset was chosen as a computationally feasible external evaluation cohort while retaining representation of both screening classes and preventing selective inclusion of favorable cases. The subset was defined before model evaluation and was used identically for all comparative models to ensure a consistent and paired evaluation protocol. For compatibility with the binary screening objective, images explicitly annotated as healthy/normal were mapped to the normal class, whereas images containing one or more annotated retinal pathological conditions were mapped to the abnormal class. No RFMiD images were used for model training, hyperparameter optimization, threshold selection, or model selection. The classification threshold was fixed at 0.50, as established during internal model development, and was applied to RFMiD without recalibration, fine-tuning, threshold adjustment, or other parameter adaptation.

### 3.3. Image Preprocessing

To minimize variation from image acquisition differences, we applied a common preprocessing pipeline to both the ODIR and BRSET development datasets while preserving the necessary diagnostic retinal information. Removal of non-retinal background areas and then resizing to 224 × 224 pixels was conducted. The RGB color representation was maintained as the input representation for the classification problem to retain chromatic and structural information related to retinal anomalies. In addition, extraction of the green color channel was done to enable anatomical and lesion-based image processing, leading to better visualization of the vascular and pathological features. Contrast Limited Adaptive Histogram Equalization (CLAHE) was used to improve the local contrast, followed by normalization of the pixel intensities to obtain a standard input range [61,62]. For the external RFMiD test dataset, limited deterministic preprocessing operations, involving resizing and normalization, were conducted. Contrast improvement and dataset-specific preprocessing steps were not conducted, thus maintaining the inherent imaging characteristics of RFMiD for independent cross-dataset evaluation.

### 3.4. Data Augmentation

Data augmentation was incorporated into the five-fold cross-validation methodology to improve training variability and reduce sensitivity to dataset-specific appearance variations [63]. Patient-level splitting was performed before data augmentation to ensure that all images for a given patient were in a single fold. During each cross-validation iteration, augmentation was applied exclusively to the four folds used for model optimization, while the remaining fold retained its original preprocessed representation. The augmentation pipeline comprised horizontal flipping, moderate rotation ($\pm 30^{\circ }$), scaling (0.9–1.1), and controlled brightness and contrast perturbations (±20%), together with mild Gaussian blur and noise. Geometric and photometric augmentation can improve robustness to variations encountered in fundus imaging [64]. MixUp (α=0.4) and CutMix (α=1.0) were additionally incorporated as regularization strategies to diversify training representations and promote smoother decision boundaries. All transformations were generated during the training process. This resulted in stochastic variations across different epochs, without permanently increasing the dataset size. RFMiD remained completely excluded from augmentation, preserving its role as an independent external cohort for cross-dataset assessment.

### 3.5. Proposed RetinoMamba-AnatomyQuery Framework

The proposed approach includes three feature-extraction pipelines, divided into two representation pathways, to overcome the lack of integration among global context, localization, and pathological data in traditional retinal classification. The first pipeline uses Vision Mamba to extract long-dependency features and global context from retinal images, producing a global representation of spatially dispersed abnormalities. The second representation pathway is made up of two specific pipelines: (i) a CNN-based feature extraction pipeline that retains detailed information about the lesion morphology and local texture, and (ii) an anatomical–lesion segmentation pipeline that offers spatial priors related to the anatomical and pathological structure of the retina. Such complementary representations are then processed by the Anatomy Query Module so that the local pathological information can be considered in light of the corresponding anatomical structure instead of appearance-based features only. Therefore, the method generates two complementary representations (global context-based and anatomy-guided local features) and combines them through feature fusion prior to binary classification, establishing a structured progression from heterogeneous retinal evidence to the final screening decision.

#### 3.5.1. Vision Mamba-Based Feature Extraction Branch

Vision Mamba serves as the global feature extraction pipeline and extracts long-range dependencies from the fundus image. This property is highly valuable in retinal screening, where disease patterns can appear in different retinal regions and depend on more general retinal features [65,66]. Vision Mamba leverages convolutional patch embedding with selective state-space modeling, providing hierarchical visual modeling with linear complexity in the number of image tokens. In addition, it employs selective state-space operations for contextual modeling, avoiding the quadratic complexity of conventional self-attention while preserving the global representation. Initially, convolutional patch embedding generates an initial feature representation for an input fundus image I, as shown in Equation (1).

(1) $$X_{0}=\phi_{emb}\left(I\right)$$

where $\phi_{emb}\left(\cdot \right)$ denotes the patch-embedding operation. The embedded features are further propagated through the Vision Mamba hierarchy, where long dependencies are modeled via selective state-space computations, with increasing levels of semantic abstraction. Equation (2) presents the process of obtaining the global retinal representation through global average pooling.

(2) $$F_{G}=\mathrm{GAP}\;\left(M\left(X_{0}\right)\right)$$

where $M\left(\cdot \right)$ denotes the hierarchical Vision Mamba transformation and $F_{G}$ represents the resulting global contextual feature vector. The resulting global contextual feature vector ${(F}_{G})$ is forwarded directly to the feature fusion stage rather than to the anatomy query module, ensuring an independent global representation of the retinal field that can be combined with the anatomy-aware local representation generated by the CNN and segmentation pathways.

#### 3.5.2. CNN-Based Local Feature Extraction Branch

To retain the fine-grained spatial details complementary to the context representation from Vision Mamba, the CNN-based feature extraction branch is employed. Retinal lesions often present themselves with significant variation in terms of their morphology, scale, and location, manifesting either as relatively small microaneurysms and exudates or larger-scale structural changes. As a result, global context modeling is insufficient for retaining the subtle local patterns when performing hierarchical feature abstraction [67]. The CNN branch can be considered a separate local representation path, which provides high-resolution morphological and texture information to the anatomy query module rather than independently contributing to the final classification decision.

EfficientNet-B3 is used as a convolutional backbone to maintain an appropriate balance between the model’s representation capacity and complexity [68]. Unlike other traditional models that mainly increase the representational power of the networks by stacking more layers or increasing network width, EfficientNet (EfficientNet-B3) uses compound scaling to balance the depth, width, and input resolution. The architecture allows for extracting hierarchical discriminative features without substantial parameters due to a significantly deeper convolutional backbone. Its application has been demonstrated in retinal fundus analysis, including multi-disease recognition and ophthalmic classification. In the proposed architecture, the model’s classification layers are removed; therefore, EfficientNet-B3 serves only as a feature extractor, avoiding an additional prediction pathway and limiting redundant computation. For an image I, local representations are extracted from selected hierarchical stages of the backbone, as expressed in Equation (3).

(3) $$F_{L}^{\left(s\right)}=E_{s}\left(I\right),\;s=1,\;\ldots ,\;S,$$

where $E_{s}$ denotes the selected EfficientNet-B3 stage and $F_{L}^{\left(s\right)}$ represents the corresponding feature map. Features extracted from shallower layers preserve spatial details, whereas features from deeper layers carry progressively more abstract pathology properties. The selected multi-scale features are mapped into a common embedding space and then fed into the anatomy query module. This proposed design differs significantly from traditional CNN-based retinal classification, where the feature maps at the deepest layer are usually pooled and mapped directly to labels. EfficientNet-B3 is explicitly separated from the classification head. The feature representations provided by EfficientNet-B3 act as the visual data that is queried by anatomically and lesion-informed queries, restricting the role of the CNN branch to feature localization, where CNN inductive biases are especially effective, while global context modeling belongs to Vision Mamba.

#### 3.5.3. Anatomical Prior Extraction Branch

The third method of feature extraction has been developed specifically to incorporate spatial information about anatomy and pathology into representation learning. Instead of treating segmentation as a separate diagnostic task, the proposed framework uses publicly available pretrained retinal segmentation models [69,70] to extract anatomical priors. The pretrained models preserve the spatial resolution of retinal structures while maintaining a low level of computational cost. Spatial information plays an especially important role in fundus image analysis, where pathology importance depends on its proximity to retinal structures. For an input fundus image I, a pretrained fundus segmentation model generates optic-disc and macular/foveal probability maps, while a pretrained U-Net model generates the retinal-vessel probability map. The parameters of these prior extractors remain frozen during training of the proposed screening framework. Their outputs are spatially aligned and used directly as anatomical prior maps for the anatomy query module, as shown in Equation (4).

(4) $$P=U\left(I\right)=\left\{P_{OD},\;P_{MF},\;P_{V}\right\}$$

where $U\left(\cdot \right)$ denotes the U-Net-based prior extraction, and the individual $P_{OD},\;P_{MF},\;and\;P_{V}$ represent the corresponding anatomical probability maps. The multi-structure extraction approach results in a structured spatial representation of the retinal region of interest, unlike using only appearance-based representations. The segmentation process serves as an intermediate step for prior extraction, rather than the final goal of this approach. The generated maps are not used as individual diagnostic results, nor are they forwarded directly to the classification subtask. They provide anatomical priors to the anatomy query module, enabling interpretation of local CNN features according to the location of their corresponding retinal structures. In this formulation, EfficientNet-B3 captures local visual appearance, while the U-Net priors provide spatial anatomical context. Their interaction through anatomy-guided query learning enables the framework to associate localized pathological evidence with relevant retinal structures before integrating it with the global Vision Mamba representation.

#### 3.5.4. Anatomy Query Module

The anatomy query module is the core methodological contribution of RetinoMamba-AnatomyQuery, transforming visual appearance cues into representations conditioned on anatomy. While existing approaches use freely initialized queries that lack explicit spatial relationships, this module constructs anatomy-guided image queries by combining anatomical probability maps with multi-scale local features extracted using EfficientNet-B3. This allows interpretation of pathology information based on its location within anatomically meaningful parts of the retina. Previous findings highlight the importance of incorporating anatomical information into query-based medical image representations [71,72], supporting an anatomy-based query formulation.

For each anatomical or pathological region k, including the optic disc, macula/fovea, and vasculature, the corresponding U-Net probability map $P_{k}$ is spatially aligned with the CNN feature map $F_{L}$. The probability map serves as a soft spatial weighting mechanism, assigning greater weight to CNN features within the relevant region. The initial query is generated as in Equation (5).

(5) $$q_{k}^{0}=W_{q}\left(\frac{\sum_{p}P_{k}\left(p\right)F_{L}\left(p\right)}{\sum_{p}P_{k}\left(p\right)+\varepsilon }\right)+e_{k},$$

where $q_{k}^{0}\in R^{256}$ denotes the initial query embedding for anatomical structure k; k represents the optic disc, macular/foveal region, and retinal vasculature; p indexes spatial positions in the local feature map; $P_{k}\left(p\right)\in \left[0, 1\right]$ denotes the anatomical prior probability for structure k at position p; $F_{L}\left(p\right)\in R^{C_{L}}$ denotes the local EfficientNet-B3 feature vector at position p; ε is a small positive constant introduced for numerical stability; $W_{q}\in R^{256\times C_{L}}$ is a learnable linear projection that maps the prior-weighted local representation to the 256-dimensional query space; and $e_{k}\in R^{256}$ is a learnable structure-specific embedding. Consequently, the weighted spatial aggregation produces a $C_{L}$-dimensional vector, which is projected by $W_{q}$ to obtain $q_{k}^{0}\in R^{256}$. The independently learnable lesion-related query is represented directly in the same 256-dimensional query space and does not require an anatomical probability map.

In parallel, a lesion-related query $\left(q_{L}^{0}\right)$ is represented as an independently learnable query embedding rather than being initialized from a lesion probability map. This query is subsequently refined together with the anatomy-guided queries through interaction with the multi-scale CNN features. Subsequently, the queries interact with multi-scale CNN features via deformable cross-attention, thereby enabling selective attention to features around anatomically important areas without exhaustive interactions. The improved queries are pooled to form the anatomy-informed representation, which is passed to the subsequent feature fusion phase.

#### 3.5.5. Anatomy-Aware Feature Fusion

The anatomy-aware feature fusion module integrates the diverse features generated from the three feature extraction pipelines while maintaining their complementary diagnostic attributes [73,74]. This process is illustrated in Figure 1, where the fusion process combines the global features $\left(F_{G}\right)$ from Vision Mamba, the local features $\left(F_{K}\right)$ from EfficientNet-B3, and the anatomy–lesion query representations $\left(Q^{*}\right)$ from the anatomy query module. The query representation contains information about the interaction between the anatomical prior $\left(P\right)$, and therefore represents local pathological information in an anatomically aware manner. Thus, the fusion process utilizes the whole-retina contextual information, local information, and anatomy-aware representations. This combination is especially important in medical imaging since local pathologies need to be understood in the broader structural context.

A fundamental challenge in the integration of the extracted feature representations arises from their heterogeneous spatial organization and feature distributions. Specifically, $F_{L}\in R^{D\times H\times W}$ preserves spatially resolved information, $F_{G}$ constitutes a global feature embedding, and $Q^{*}\in R^{K\times D}$ comprises *K* refined anatomy query tokens. During standard fusion, these representations may introduce dimensional and scale inconsistencies and may disproportionately emphasize features with larger activation magnitudes. Therefore, using Equation (6), dimensional alignment and feature normalization are performed.

(6) $${\bar{F}}_{L}=LN\left(GAP\left(F_{L}\right)\right),\;\;F_{Q}=LN\left(POOL\left(Q^{*}\right)\right),\;\;{\bar{F}}_{G}=LN\left(F_{G}\right),$$

where $GAP\left(.\right)$ denotes global average pooling, $POOL\left(.\right)$ aggregates the refined query tokens, and $LN\left(.\right)$ indicates layer normalization. The outputs from the heterogeneous components are made compatible for scaling without changing their semantic characteristics. The learnable linear projections project the normalized features to a shared latent space, thereby ensuring consistency for interaction.

Following representation alignment, the normalized global and local features are concatenated and projected to obtain the contextual appearance representation $\left(F_{c}\right)$. The query-derived representation $F_{Q}$ is subsequently employed as an anatomy-conditioned modulation signal. Equation (7) expresses the proposed fusion strategy.

(7) $$F_{c}=W_{c}\left[{\bar{F}}_{G}∥{\bar{F}}_{L}\right]+b_{c},\;\;F_{F}=F_{c}+\phi \left[\gamma \left(F_{Q}\right)⊙F_{c}+\beta \left(F_{Q}\right)\right],\;$$

where ${\bar{F}}_{G}\in R^{256}$ and ${\bar{F}}_{L}\in R^{256}$ denote the normalized global and pooled local representations, respectively; ∥ denotes feature concatenation; $W_{c}\in R^{256\times 512}$ and $b_{c}\in R^{256}\;$ are the learnable projection matrix and bias; and $F_{c}\in R^{256}$ is the resulting combined representation. $F_{Q}\in R^{256}$ denotes the pooled representation of the refined anatomy- and lesion-related queries. The learnable transformations γ(.) and β(.) map $F_{Q}$ to 256-dimensional feature-wise scaling and shifting vectors, respectively. The symbol ⊙ denotes element-wise multiplication, while *ϕ* is a learnable scalar controlling the strength of anatomy-conditioned modulation. The final fused representation $F_{F}\in R^{256}$ therefore retains the same dimensionality as $F_{c}$, ensuring dimensional consistency throughout the fusion operation. The anatomy-conditioned representation is more than a mere concatenated feature vector. It actively controls how each dimension contributes to the global–local representation, enabling specific adjustment of the fused feature representation according to the anatomical/pathological information encoded through the refined queries.

The residual formulation in Equation (7) provides a well-defined procedure for preserving information in adaptive fusion. While the identity path preserves the original contextual information, query-conditioned information selectively enhances or reduces diagnostic feature dimensions. Therefore, features with minimal modulation are not necessarily excluded, which matters especially for heterogeneous retinal abnormalities, since their appearances may fall outside the representative anatomical or lesion patterns learned by the queries. This framework differs from typical concatenation or element-wise fusion, where heterogeneous representations are combined without contextual control, and from fixed-weight fusion, where each representation’s weight is constant across all inputs. Additionally, anatomy-aware feature fusion improves representation learning by emphasizing discriminative information while preserving complementary information.

Anatomy-aware feature fusion can be viewed as a fusion of dimensionally aligned global and local representations conditioned on anatomical context, along with a residual representation of complementary evidence. The modulation coefficients used in this approach are jointly optimized with the classification task rather than the pre-determined branch-specific weights, adjusting the relative importance of different feature dimensions according to the anatomy of individual samples. The representation preserves global context, localized appearance information, and anatomy-specific pathologies within a discriminative space, establishing a structured transition from heterogeneous feature extraction to anatomically informed decision representation while minimizing the loss of diagnostically relevant information during fusion.

During the fusion process, computational efficiency is further maintained by aligning the heterogeneous representations within a common latent dimension before applying anatomy-conditioned modulation. The proposed FiLM-based operation performs feature-wise scaling and shifting using compact query-derived parameters, avoiding computationally intensive pairwise attention across all feature tokens. Residual preservation additionally retains the contextual representation without requiring a separate reconstruction pathway. Thus, adaptive anatomical conditioning adds limited operations while preserving the original fused representation.

#### 3.5.6. Classification Head

The classification head transforms the anatomy-aware fused representation $F_{F}$ into the final binary retinal screening decision. As $F_{F}$ consolidates global contextual information, fine-grained local characteristic, and anatomy-conditioned evidence, a lightweight prediction head is employed to preserve the discriminative structure established during feature fusion without introducing unnecessary parameterization. As shown in Equation (8), dropout regularizes the fused representation, which is then projected onto a scalar classification logit.

(8) $$z=W_{cls}Dropout\left(F_{F}\right)+b_{cls},\;\hat{P}=\sigma \left(z\right)=\frac{1}{1+e^{-z}},$$

where $W_{cls}$ and $b_{cls}$ denote the learnable classification parameters, z represents the unnormalized decision score, and $\sigma \left(.\right)$ denotes the sigmoid activation function, making binary classification. The resultant probability quantifies the likelihood that the input fundus image belongs to the abnormal class, whereas 1-$\hat{P}$ represents the corresponding probability of the normal class. Equation (9) computes the final screening decision $\left(\hat{y}\right)$ using a predefined decision threshold $\left(\tau \right)$.

(9) $$\hat{y}=\left\{\begin{array}{c} 1\left(Abnormal\right),\;\hat{P}\geq \tau \;\\ 0\left(Normal\right),\;\hat{P}<\tau \; \end{array}\right.$$

Thus, the classifier head primarily acts as a decision-mapping layer, ensuring a direct relationship between the anatomical information-driven fusion feature and the inferred screening result. The class score output is used to form the target signal for the explanation procedure described in the subsequent section.

The classification head remains a lightweight terminal decision component, as the preceding global, local, and anatomy-conditioned pathways perform the principal representation learning. Following dropout regularization, the fused representation $F_{F}$ is projected directly onto a scalar binary classification logit, reducing additional parameters at the prediction stage and keeping computational complexity concentrated in diagnostically meaningful representation learning rather than an unnecessarily deep classification head.

#### 3.5.7. Grad-CAM++-Based Explainability of the Fused Decision

Following binary classification, Grad-CAM++ is used as a post hoc attribution method to examine the spatial evidence associated with the final normal/abnormal decision. The classification score originates from $F_{F}$, integrating of global features $\left(F_{G}\right)$, local features $\left(F_{L}\right)$, and anatomy-conditioned query information $\left(Q^{*}\right)$. However, the dimensional alignment and pooling operations preceding fusion transform these representations into compact embeddings, and $F_{F}$ consequently lacks the two-dimensional spatial organization required to construct a retinal activation map. Spatial attribution is therefore anchored to $F_{L}$, which retains the *H* × *W* organization of the retinal representation while remaining computationally connected to the final fused prediction. Gradient-based class-activation mapping derives spatial explanations by relating the target class score to spatially resolved feature maps. For a target class *c*, the attribution procedure is represented as shown in Equation (10).

(10) $$H_{GC++}^{c}=GradCAM++\left(F_{L},\frac{\partial z^{c}\left(F_{F}\right)}{\partial F_{L}}\right)$$

where $z^{c}\left(F_{F}\right)$ denotes the final logit associated with class *c*, and $H_{GC++}^{c}$ represents the resulting class-specific attribution map. As shown in Equation (10), attribution gradients depend on the classifier’s output score and propagate backward to the spatial representation through the classification head and AAF module. Thus, the activation map reflects retinal areas whose local representations influenced the final decision through the entire downstream fusion process, rather than an independent prediction from the EfficientNet-B3 branch. This formulation maintains a strict separation between decision-making and decision explanation. The fusion representation performs only classification, while CNN-based representations provide the coordinate system to localize decision-related evidence on the fundus image. Consequently, Grad-CAM++ maps are interpreted as spatial attribution of the fused classification decision through the local feature pathway, rather than as a complete decomposition of each feature’s individual contribution. This interpretation is particularly important for a multi-branch architecture, as a single two-dimensional activation map cannot independently infer non-spatial global and query representations. Grad-CAM++ facilitates qualitative analysis of how much the decision-relevant spatial evidence matches clinically relevant retinal regions.

### 3.6. Optimization Objective

The proposed RetinoMamba-AnatomyQuery framework was optimized using a joint objective comprising binary classification loss and query-diversity regularization. Binary cross-entropy was employed as the primary classification loss to optimize discrimination between normal and abnormal fundus images and was assigned unit weight. In parallel, query-diversity regularization was introduced to encourage the refined anatomy- and lesion-related queries to capture complementary information and avoid convergence toward highly similar representations. The regularization penalizes excessive cosine similarity between pairs of refined query embeddings when their similarity exceeds a predefined margin of 0.20.

The contribution of query regularization was controlled by a weighting coefficient. Candidate weights of 0.01, 0.05, 0.10, and 0.20 were evaluated using the validation folds while maintaining identical training settings. A weight of 0.05 provided the most favorable validation performance while preserving stable query diversity and was therefore fixed for all subsequent experiments. The anatomical prior extractors remained frozen throughout screening-model training; consequently, no segmentation loss was included in the optimization objective. This design maintains binary retinal screening as the principal learning objective while allowing query regularization to promote complementary anatomy-aware representations without introducing an additional inference-stage operation.

The primary classification loss, *L*_cls_, optimizes discrimination between normal and abnormal fundus images. The auxiliary query loss, *L*_query_, promotes consistency between the refined anatomy–lesion queries and their corresponding prior representations. Equation (11) describes the training objective for the proposed model development.

*L*_total_ = *L*_cls_ + *λ*_q_
*L*_query_, (11)

where *λ*_q_ are weighting coefficients that regulate the contributions of the segmentation and query-consistency objectives, respectively. This joint optimization keeps the classification task as the primary learning objective while anatomical and lesion-related supervision provides complementary structural guidance during training. This formulation keeps classification as the central learning task while letting anatomical supervision guide representation learning without disrupting the overall optimization process. Joint classification and segmentation supervision has shown its usefulness in training complementary representations in the medical imaging domain. The auxiliary objectives are confined to the optimization process, without adding any loss-dependent steps during classification inference.

### 3.7. Experimental Evaluation Strategy

The experimental protocol was designed to evaluate discrimination, robustness, generalizability, and computational efficiency under controlled conditions. Model development employed patient-level five-fold cross-validation, maintaining complete patient separation between folds to prevent information leakage. Performance was quantified using accuracy, recall, specificity, precision, F1-score, Matthews correlation coefficient (MCC), Cohen’s κ (Kappa), Area Under the Receiver Operating Characteristic Curve (AUROC), and Area Under the Precision–Recall Curve (AUPRC), with 95% confidence intervals (CIs) reported where appropriate. Generalizability is independently assessed on RFMiD without retraining or parameter adaptation. Comparative architectures were reimplemented using the harmonized binary endpoint and identical patient-level partitions to minimize dataset- and protocol-related bias. Statistical comparisons were based on pooled out-of-fold predictions [75]. For each model, predictions generated exclusively on the held-out portion of each of the five patient-level folds were concatenated to form a complete out-of-fold prediction vector. Predictions from different models were then matched using identical patient/image identifiers. The same patient-level fold assignments were maintained across all models, ensuring that each paired observation represented predictions for the same sample under equivalent evaluation conditions. No training-fold predictions were included in the statistical comparisons. Ablation experiments quantify the individual contributions of global modeling, local representation, anatomy-query learning, and anatomy-aware fusion, while parameter count, floating-point operations (FLOPs), and inference time characterized computational complexity.

## 4. Results

We conducted experiments using the standardized experimental configuration in Table 1, which defines the computation environment, architectural settings, optimization parameters, and inference conditions used throughout the study. RetinoMamba-AnatomyQuery was designed to achieve a favorable trade-off between representational power and computational efficiency while retaining its multi-pathway structure. A linear-complexity state-space model provides global representation, efficient convolutional processing captures local retinal features, compact query embeddings encode anatomical features, and low-dimensional FiLM transformations enable efficient cross-modal interaction. The approach ensures computational efficiency while enabling interactions among global, local, and anatomical representations. Further analyses systematically assess predictiveness, generalization, component-level performance, computational efficiency, and decision interpretability.

Table 2 reports that RetinoMamba-AnatomyQuery achieved consistently high discriminative performance across the five folds of patient-level cross-validation, with small inter-fold variance in all evaluation metrics. The model achieved mean accuracy, recall, specificity, and F1-score of 97.93%, 97.77%, 98.07%, and 97.82%, respectively, indicating balanced discrimination between normal and abnormal fundus images. Standard Vision Mamba served as the main reference baseline, representing the effect of global state-space modeling alone, and provided an architecture-specific baseline for evaluating the proposed framework’s incremental contribution. Vision Mamba has shown its ability to learn global representations in fundus-image analysis. Compared with Vision Mamba, RetinoMamba-AnatomyQuery improved principal classification metrics by more than 2.5%. Sensitivity and specificity improvements indicate that the improvements are consistent for detecting abnormal cases as well as identifying normal cases. MCC and Cohen’s κ additionally proved high consistency and significant agreement. The statistical significance of the improvement was tested via McNemar’s test on paired patient-level predictions out of fold at a two-sided significance level of *p* < 0.05. Collectively, these findings indicate that integrating local retinal characteristics and anatomy-conditioned representations with Mamba-derived global context provides measurable discriminative value beyond standard Vision Mamba.

Table 3 presents the ablation results evaluating the importance of each RetinoMamba-AnatomyQuery component. Standard Vision Mamba serves as the benchmark for the global contextual modeling framework. Standard Vision Mamba serves as a base model for global contextual modeling. Adding the EfficientNet-B3 component, responsible for local feature extraction, boosts performance by combining long-range retinal context with rich spatial features. This finding is consistent with the recent research showing that CNN–Mamba integration effectively incorporates both local feature extraction and global contextual modeling capability [59]. Including anatomical and lesion priors in the next step further increases discriminatory power, indicating the effectiveness of explicitly incorporating structurally relevant information into the learning process.

The anatomy query module yields a more significant improvement, suggesting that guided interaction using retinal anatomy knowledge is more beneficial than the traditional combination of visual features. The anatomy-aware feature fusion module provides a better representation by enabling adaptive feature-wise modulation, allowing anatomical information to control the integration of global and local features. The residual pathway preserves complementary discriminative information that is not affected by anatomical information. Adaptive integration of heterogeneous global and local representations was shown to provide additional feature complementarity in medical imaging. The proposed approach achieves the best results on all reported classification metrics, demonstrating the cumulative effect of global contextual modeling, localized feature extraction, anatomy-guided query learning, and adaptive anatomy-aware fusion.

**Figure 2:**
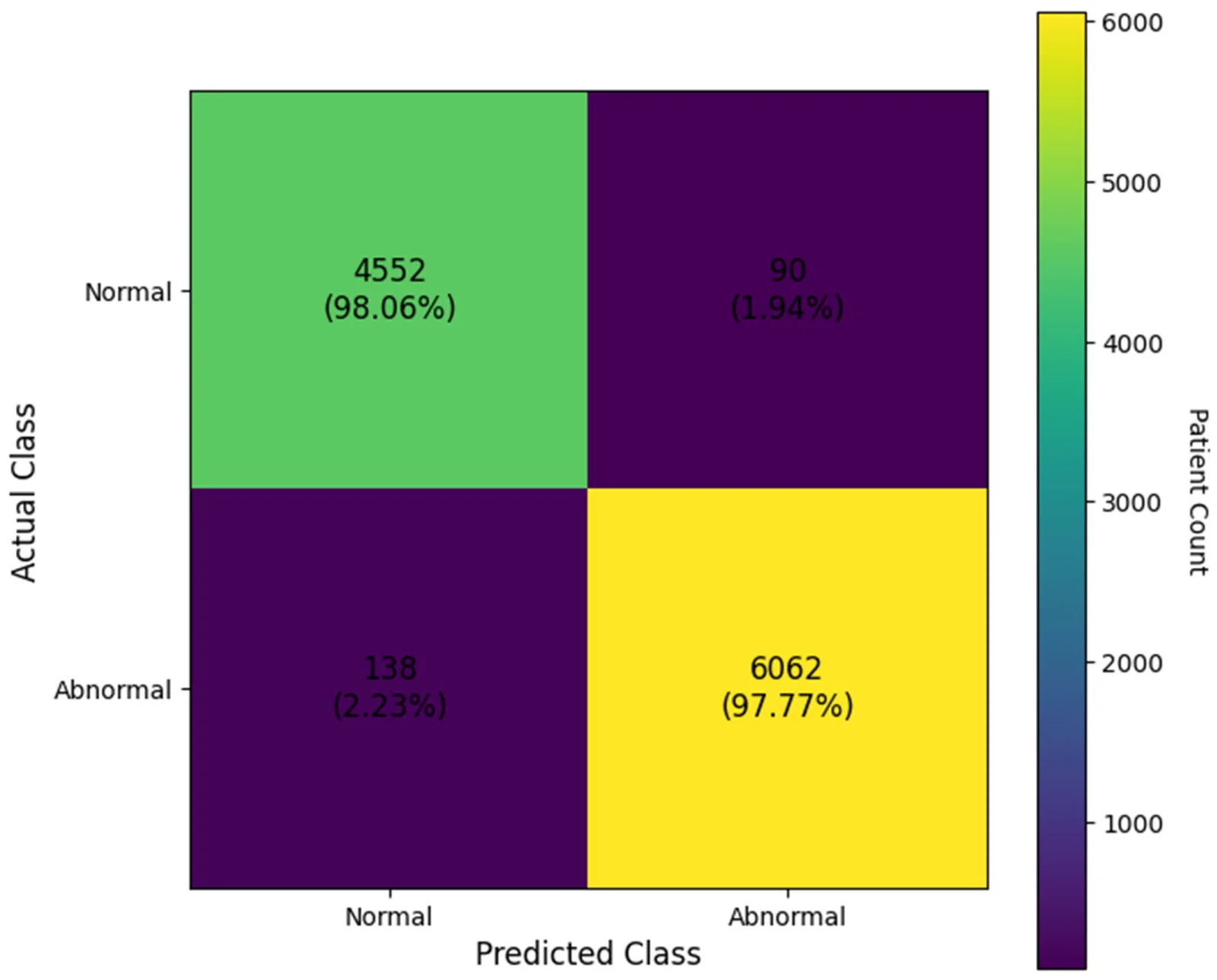
The patient-level pooled out-of-fold confusion matrix (internal dataset).

Figure 2 shows the pooled patient-level confusion matrix for RetinoMamba-AnatomyQuery across the complete five-fold cross-validation procedure. In each fold, about one-fifth of the total patient set formed the held-out evaluation set, whereas the remaining folds were used to develop the classifier. After all five iterations, we collected predictions made only on the corresponding held-out sets to form the pooled out-of-fold confusion matrix. This means each patient had only one prediction, ensuring no patient was evaluated multiple times; thus, no single fold was preferred over the others. From the results shown in Figure 2, of the 6200 abnormal patients, 6062 were correctly predicted as abnormal, yielding a sensitivity of 97.77%, whereas 138 were falsely predicted as normal. In the 4642 normal patient pool, 4552 were correctly predicted to be normal, yielding a specificity of 98.06%. The few false negatives and false positives suggest balanced discrimination between both classes. The minor discrepancy between the pooled confusion-matrix accuracy and the mean fold-wise accuracy arises from pooling independent out-of-fold predictions at the cohort level and taking the arithmetic mean of fold-specific accuracies.

**Figure 3:**
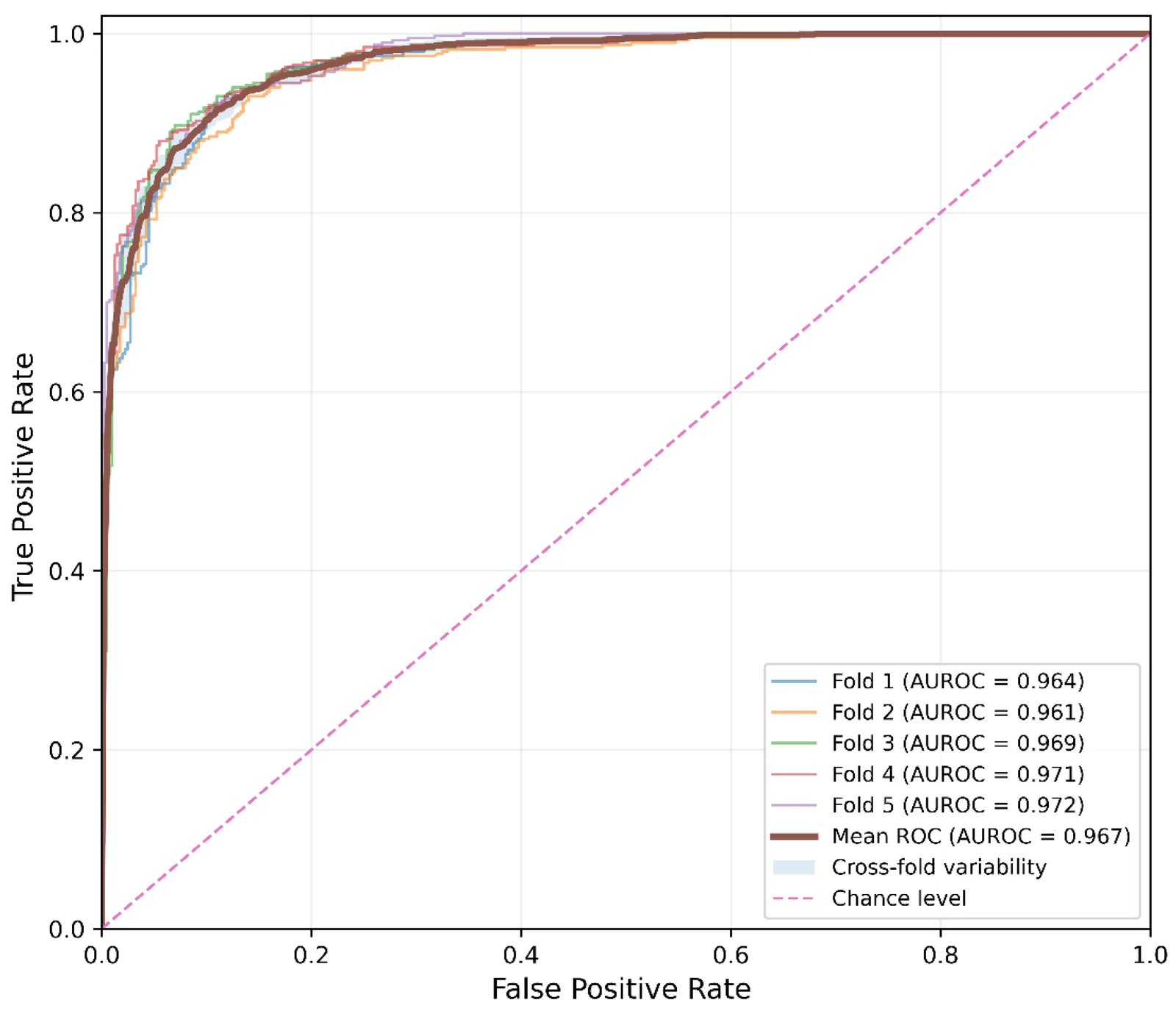
ROC analysis of the proposed RetinoMamba-AnatomyQuery-based binary retinal screening.

Figure 3 demonstrates the ROC analysis for RetinoMamba-AnatomyQuery on five patient-wise cross-validation folds. The consistently steep slopes toward the upper-left region indicate effective differentiation between normal and abnormal fundus images across different decision boundaries. The AUROC scores range from 0.961 to 0.972, with an average AUROC of 0.967, indicating consistent performance across patient-wise datasets. The minimal variance in performance among different cross-validation folds further emphasizes the effectiveness of the learned representation. The significant differentiation is due to the combined use of retinal context, pathology, and anatomical features.

**Figure 4:**
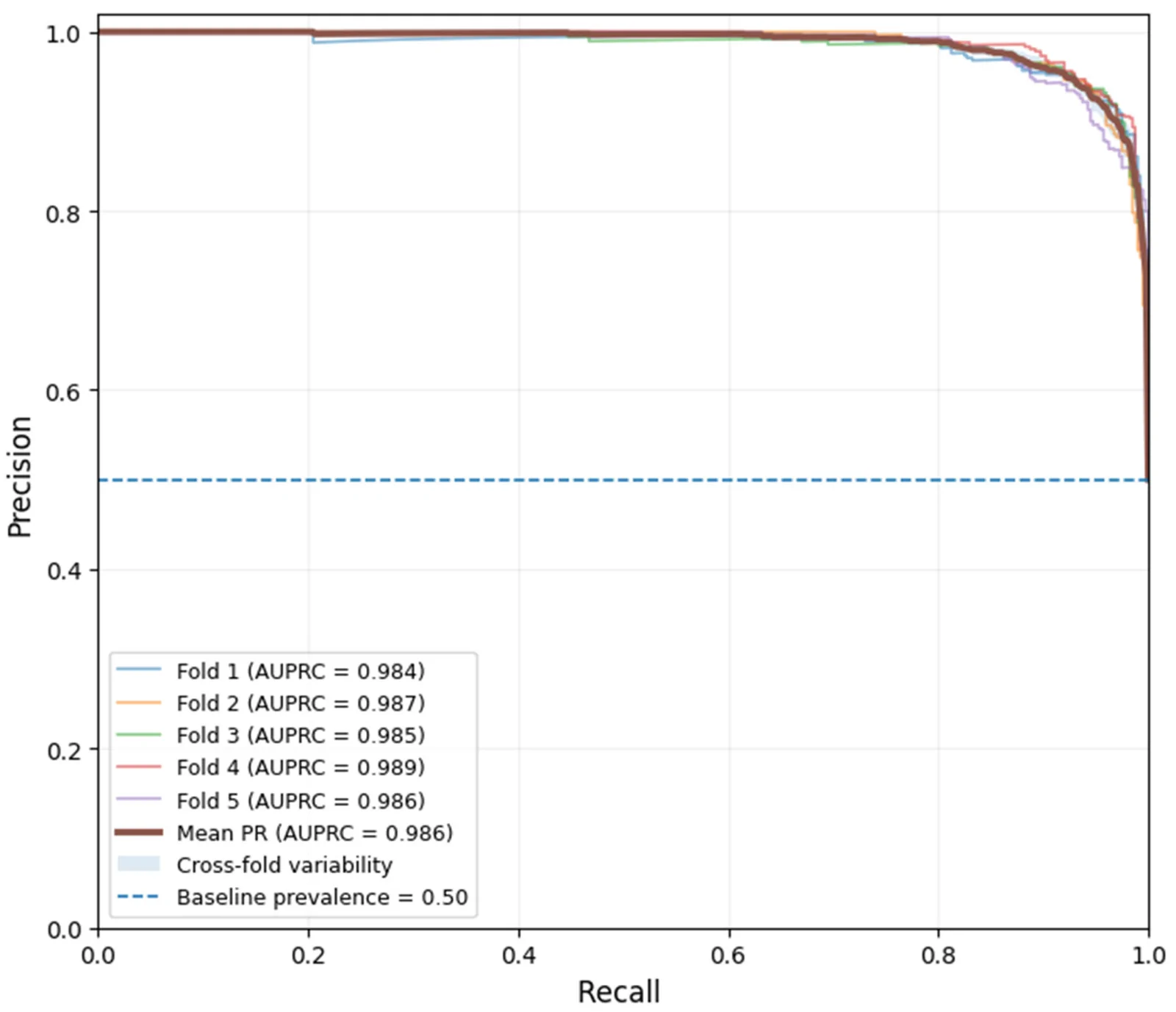
Five-fold precision–recall analysis of RetinoMamba-AnatomyQuery for abnormal fundus detection.

Figure 4 shows the precision–recall characteristics for RetinoMamba-AnatomyQuery, illustrating the system’s ability to classify abnormal fundus examinations while controlling false-positive rates. The AUPRC scores across folds ranged from 0.984 to 0.989, with an average of 0.986, substantially exceeding the reference value of 0.50. The high area-under-the-curve values indicate high precision regardless of recall, meaning higher recall is achieved with almost no loss in prediction quality.

The selected comparative models [46,47,48,49,55] represent distinct contemporary learning architectures for binary classification of color fundus photographs using transfer-learning CNNs, generalized fundus-image models, Vision Transformers with masked autoencoding, and Vision Mamba. Although these models originated with disease-specific binary classification endpoints (for example, diabetic retinopathy or glaucoma), they may be considered reference models to assess whether various representation-learning techniques can effectively classify normal-versus-abnormal retinal images. The performance metrics from the original studies for quantitative comparison are not used due to cross-study variability in dataset composition, disease prevalence, image acquisition and processing, partitioning strategy, and evaluation approach, which introduce bias and obscure true architectural improvements. In addition, changes in data partitioning alone may result in noticeable differences in performance metrics.

In order to construct a fair comparison between models, the reference architectures were reimplemented and retrained on the same development dataset using an identical normal-versus-abnormal classification endpoint. All models were tested using the five-fold patient-level partitioning of the development dataset. This harmonization reduces methodological and dataset-related confounding, thereby providing a more appropriate basis for attributing observed performance differences to the underlying representation-learning strategies.

Furthermore, the trained models were evaluated on the external independent dataset without any retraining or parameter tuning. This allows for evaluation of whether performance differences observed on the development set persist under distribution shift rather than being driven by adaptation to the development dataset. External evaluation is particularly important in medical AI, where models developed on a single data source can exhibit substantial performance degradation when transferred to independent populations. Overall, harmonized retraining and common external evaluation establish a controlled, architecture-centered comparative framework while avoiding potentially misleading comparisons of performance values from heterogeneous studies.

Table 4 compares the proposed RetinoMamba-AnatomyQuery with the reimplemented models using the same patient-level five-fold validation. In terms of performance metrics, RetinoMamba-AnatomyQuery achieves the best results, with an accuracy of 97.93%, recall of 97.77%, specificity of 98.07%, precision of 97.87%, and F1-score of 97.82%. The Vision Mamba model architecture (Vim-Small variant), derived from Ferreira et al. [55], achieved the best baseline performance, with 95.06% accuracy and a 94.84% F1-score. The RetinoMamba-AnatomyQuery model outperformed this benchmark model in accuracy and F1-score by 2.87% and 2.98%, respectively. The performance gain was consistent across recall, specificity, and precision, suggesting that the model performs better at discriminating between normal and abnormal classes.

Table 5 depicts a multi-metric comparison between the proposed RetinoMamba-AnatomyQuery and state-of-the-art models on an independent external dataset. We evaluated the models on 20% of the RFMiD dataset without training, tuning, or parameter adjustments to measure their ability to transfer to an out-of-sample distribution of fundus images. The performance on the external set was measured in terms of image-level performance rather than patient-level performance due to the lack of a method to identify patients reliably within the RFMiD dataset. Thus, performance on RFMiD demonstrates image-level generalization across datasets, whereas performance on the five-fold internal test set represents the patient-level performance measure. RetinoMamba-AnatomyQuery achieves accuracy, recall, specificity, precision, and F1-scores of 96.21%, 95.86%, 96.52%, 96.13%, and 95.99%, respectively. The Standard Vision Mamba is the best comparator but is consistently outperformed by the framework across the entire performance metrics. Recall and specificity improvements simultaneously suggest that the model improves the identification of abnormal cases without affecting the reliable detection of normal cases.

As shown in Table 6, RetinoMamba-AnatomyQuery retains a positive trade-off between prediction accuracy and computational cost. The framework consists of 23.9 million parameters and 8.3 billion FLOPs, placing the computation complexity of the framework in the interval typical for compact architectures. While the framework employs global, local, and anatomy-guided representations, it achieves an inference time of 14.5 ms per image and uses a peak GPU memory of 2.7 gigabytes. These values remain considerably lower than the computational demands of the ViT-based configuration and competitive with the CNN- and Mamba-based alternatives. The results demonstrate that the proposed architectural integration introduces limited computational overhead while maintaining efficient inference, supporting a favorable performance–complexity trade-off for retinal screening.

Table 7 presents additional evidence on the spatial aspect of predictions generated by RetinoMamba-AnatomyQuery. The findings show that activation patterns differ between the two groups, with high-intensity regions corresponding to retinal areas that play a more significant role in generating the respective decision. Importantly, the activations obtained are mostly found in the retinal field as well as in the areas with an anatomical or pathological meaning as compared to those located in the non-informative image background. The results provide qualitative evidence to support ophthalmologists in making effective decisions.

## 5. Discussions

The experimental results demonstrate that anatomical representation learning provides an efficient approach to automating retinal image processing when the task involves distinguishing normal retinal tissue from various types of pathology. The consistently high predictive performance on both internal and external datasets indicates that the obtained feature representations capture inherent retinal properties rather than imaging-specific artifacts that would otherwise reduce generalization. Notably, limited generalization remains a key challenge for deep neural network-based models in real-world medical applications. Imaging modality differences, changes in imaging parameters, lighting conditions, and demographic factors can induce domain shift, reducing model performance. The model’s stable predictive accuracy across domains indicates that incorporating anatomical knowledge into the representation learning process improves feature generalizability and stability across imaging conditions. Unlike the traditional approach, which focuses on appearance-based discrimination, the proposed learning framework allows the model to preserve structural consistency while adapting the representation to diverse pathological appearances.

The findings validate the importance of anatomical context as an essential element of retinal image interpretation. Retinal pathologies rarely appear in isolation, and their diagnostic significance is determined by their spatial relationships with adjacent anatomical structures, such as the optic disc, macula, fovea, and retinal vascular system. Although many studies have used anatomical information for retinal analysis via segmentation-assisted learning, anomaly identification, or graph-based representations, they mostly used anatomical knowledge as an additional supervisory signal rather than as an integral part of feature representation learning. This improvement suggests that integrating anatomical priors into representation learning yields more informative representations that preserve structural consistency while accommodating pathologies. Thus, the study’s findings extend understanding of anatomical information as an essential part of representational learning, rather than merely an auxiliary classification tool. This aligns with clinical practice, where the eye’s anatomical structure provides context for interpreting retinal pathologies; thus, the results support the broader scientific view that anatomical representation learning is a promising direction for developing explainable artificial intelligence in ophthalmology.

The primary goal of any clinical screening task is to detect retinal images that require additional examination rapidly. Therefore, in routine screening, binary retinal screening is the first step in the clinical decision-making pipeline, with a preference for reliable decisions for diagnosing a particular eye disease. Grouping different retinal diseases into a single pathological category allows the framework to identify general patterns in the retina, reducing the impact of class ambiguity on performance, which often occurs in multi-class classification tasks. This feature makes the suggested binary retinal screening a useful tool for large-scale and population screening studies, where efficiency, scalability, and rapidity matter more than immediate disease diagnosis. Moreover, the framework’s performance on independent data samples indicates its potential applicability to diverse clinical environments with varying image acquisition device characteristics and patient populations. The proposed model serves as a clinical decision support system that enables early detection of retinal abnormalities and improves referral processes.

Nonetheless, the current study has a few limitations that should be considered when interpreting its results. Although the binary screening task reflects the initial screening stage used in clinical retinal programs, it does not allow for distinction of specific diseases or severity levels. The proposed model is validated using public datasets, which may not capture the variability in imaging devices, ethnic composition, healthcare infrastructure, and disease prevalence found in clinical practice. The use of anatomical supervision in the learning procedure assumes reliable auxiliary annotations. Although techniques for automatic anatomical segmentation are rapidly evolving, variations in segmentation quality can affect learning-process results. Finally, the scope of the current work does not include prospective validation in routine clinical workflows, so further research is needed to deploy the proposed framework in clinical practice.

To address these limitations, the following research directions are suggested. Multi-class retinal disease classification tasks could be investigated using anatomy-aware representation learning to detect retinal pathologies simultaneously while maintaining the described advantage of anatomic consistency. Additionally, applying self-supervised and foundation model learning techniques could improve efficiency and generalizability, reducing dependence on manually labeled data while preserving the transferability of learned representations across ophthalmic datasets. Combining complementary modalities, such as optical coherence tomography, fluorescein angiography, and ultra-widefield retinal imaging, may facilitate the development of multimodal anatomical representations for detailed characterization of retinal disorders. Finally, introducing uncertainty quantification, domain adaptation, and continual and federated learning approaches may improve the efficiency and reliability of the learning algorithm and make it ready for application in geographically distributed healthcare facilities while preserving patients’ privacy.

## 6. Conclusions

This study shows that complementary representation learning can achieve effective retinal abnormality screening without increasing architectural scale. The proposed framework establishes anatomy-conditioned integration as a central mechanism for reconciling whole-retina context with localized pathological evidence. The ablation findings reinforce this contribution, showing that progressively incorporating local representation, anatomical priors, query refinement, and adaptive fusion yields complementary improvements beyond the global backbone alone. Comparative evaluation further indicates that these architectural contributions yield stronger discrimination while maintaining a controlled computational profile. The study has broader implications for automated fundus screening. A normal/abnormal decision can serve as an efficient first-stage triage mechanism, reducing the complexity of direct multiclass prediction when the immediate objective is to identify examinations requiring further clinical attention. The proposed approach therefore represents a foundation for computationally efficient screening rather than a substitute for definitive ophthalmological diagnosis. Several limitations define the scope of the present findings. The retrospective nature of the datasets restricts conclusions regarding prospective clinical effectiveness. External evaluation was necessarily conducted at the image level, as reliable patient identifiers were unavailable in RFMiD. Variations in imaging devices, acquisition conditions, population characteristics, and disease prevalence may introduce additional distribution shifts not fully represented in the current evaluation. Furthermore, Grad-CAM++ provides supportive spatial attribution and should not be interpreted as a complete explanation of model reasoning. Future investigations should prioritize prospective multicenter validation, patient-linked external cohorts, device-independent robustness, calibration and uncertainty estimation, and clinically defined referral thresholds. A subsequent extension toward hierarchical screening, in which detected abnormalities are further differentiated into disease-specific categories, could establish a more comprehensive pathway from automated triage to clinically informative retinal decision support.

## Figures and Tables

**Table 1:** Implementation configuration of the proposed RetinoMamba-AnatomyQuery framework.

Category	Configuration	Implementation Details
Computing platform	Operating system	Windows 11 (64-bit)
	GPU	NVIDIA GeForce RTX 4090, 24 GB VRAM
	CPU	Intel Core i9
	System memory	64 GB RAM
	GPU acceleration	CUDA 12.1, cuDNN 8.9
Software environment	Programming language	Python 3.10
	Deep learning framework	PyTorch 2.1
	Supporting libraries	torchvision, OpenCV, Pillow, NumPy, SciPy, scikit-learn
Input configuration	Image type	Retinal fundus image
	Input resolution	(224 × 224) pixels
	Input channels	3
	Tensor dimension	(3 × 224 × 224)
	Normalization	Channel-wise intensity normalization
Global feature extraction	Backbone	Vim-Tiny
	Modeling mechanism	Selective state-space modeling
	Function	Long-range retinal contextual representation
	Output representation	(*F*_G_)
	Projected feature dimension	256
Local feature extraction	Backbone	EfficientNet-B3
	Classification layer	Removed
	Function	Fine-grained spatial retinal feature extraction
	Output representation	(*F*_L_ ∈ ℝ^D×H×W^)
	Aligned feature dimension	256
Anatomical prior extraction	Architecture	Frozen pretrained retinal segmentation (U-net) models
	Function	Anatomical prior extraction
	Output	Optic-disc, macular/foveal, and vessel probability maps
	Downstream connection	Anatomy query module
Anatomy query module	Query initialization	Anatomy-guided initialization; independently learnable lesion-related query.
	Query interaction	Deformable cross-attention between anatomy/lesion-related queries and multi-scale local features.
	Query dimension	256
	Refined queries	(Q* ∈ ℝ^4×256^)
	Query aggregation	Pooling followed by layer normalization
	Aggregated representation	(*F*_Q_ ∈ ℝ^256^)
Feature alignment	Local feature aggregation	Global average pooling
	Feature normalization	Layer normalization
	Common latent dimension	256
Anatomy-aware fusion	Contextual fusion	Concatenation and learnable linear projection of (*F*_G_) and (*F*_L_)
	Anatomical conditioning	(*F*_Q_)-conditioned FiLM
	Modulation	Learnable feature-wise scaling (γ) and shifting (β)
	Feature preservation	Residual connection
	Final fused representation	(*F*_F_ ∈ ℝ^256^)
Classification head	Regularization	Dropout = 0.30
	Prediction layer	Single fully connected output
	Activation	Sigmoid
	Decision threshold	0.50
	Output classes	Normal/abnormal
Optimization	Optimizer	AdamW
	Initial learning rate	(1 × 10^−4^)
	Weight decay	(1 × 10^−4^)
	Batch size	16
	Maximum epochs	100
	Learning-rate scheduler	Cosine annealing
	Classification loss	Binary cross-entropy
	Overall objective	(ℒ_cls_ + λ_q_ℒ_query_)
Training configuration	Precision	Automatic mixed precision (FP16/FP32)
	Random seed	42
	Model selection	Lowest optimization/validation loss within each fold
	Internal evaluation	Patient-level five-fold cross-validation
	External evaluation	Independent external testing without retraining
Explainability	Method	Grad-CAM++
	Attribution target	Final classification score
	Spatial reference	(*F*_L_)
	Application	Post hoc visualization

**Table 2:** Five-fold cross-validation performance of RetinoMamba-AnatomyQuery and comparison with the best-performing baseline.

Metric	Fold 1	Fold 2	Fold 3	Fold 4	Fold 5	Proposed Mean ± SD	Standard Vision Mamba Baseline (Vim-Tiny Baseline)
Accuracy (%)	97.72	98.08	97.84	98.16	97.85	97.93 ± 0.17	95.18
Recall (%)	97.51	97.86	97.63	98.01	97.82	97.77 ± 0.19	94.86
Specificity (%)	97.91	98.27	98.02	98.29	97.88	98.07 ± 0.19	95.42
Precision (%)	97.68	98.03	97.81	98.09	97.76	97.87 ± 0.18	95.09
F1-score (%)	97.59	97.94	97.72	98.05	97.79	97.82 ± 0.18	95.01
MCC	0.954	0.962	0.957	0.964	0.957	0.959 ± 0.004	0.913
Cohen’s κ	0.954	0.962	0.957	0.964	0.957	0.959 ± 0.004	0.912

**Table 3:** Ablation analysis of individual components in the proposed RetinoMamba-AnatomyQuery model.

Configuration	$F_{G}$	$F_{L}$	$P_{k}$	$Q^{*}$	AAF/FiLM	Residual	Accuracy (%)	Recall (%)	Specificity (%)	Precision (%)	F1-score (%)	Params (M)	FLOPs (G)
A1: Standard Vision Mamba	✓	–	–	–	–	–	95.18	94.86	95.42	95.09	95.01	6.8	1.5
A2: CNN local branch	–	✓	–	–	–	–	94.82	94.41	95.19	94.68	94.63	17.2	5.8
A3: Global + local	✓	✓	–	–	–	–	96.11	95.87	96.32	96.09	95.98	20.6	6.6
A4: + Anatomy/lesion priors	✓	✓	✓	–	–	–	96.54	96.28	96.77	96.55	96.40	22.9	7.6
A5: + Anatomy query module	✓	✓	✓	✓	–	–	97.10	96.84	97.34	97.08	96.96	23.4	7.9
A6: + Anatomy-aware FiLM fusion	✓	✓	✓	✓	✓	–	97.52	97.26	97.76	97.51	97.38	23.7	8.1
A7: Full proposed framework	✓	✓	✓	✓	✓	✓	97.93	97.77	98.07	97.87	97.82	23.9	8.3

Note: $F_{G}$, $F_{L}$, $P_{k}$, and $Q^{*}$ denote the global Vision Mamba representation, local CNN representation, anatomy/lesion priors, and refined anatomy queries, respectively. AAF denotes anatomy-aware feature fusion and FiLM represents Feature-wise Linear Modulation.

**Table 4:** Patient-level five-fold comparative performance on the internal dataset.

Model	Accuracy, % (95% CI)	Recall (%)	Specificity (%)	Precision (%)	F1-Score (%)	MCC	Cohen’s κ
Abramovich et al. (2025) [46]	94.82 (94.10–95.49)	94.51	95.13	94.76	94.63	0.896	0.896
Yang et al. (2024) [47]	95.21 (94.55–95.82)	94.93	95.47	95.16	95.04	0.904	0.904
Chaurasia et al. (2025) [48]	94.96 (94.27–95.59)	94.64	95.25	94.89	94.76	0.899	0.899
Saproo et al. (2024) [49]	94.37 (93.62–95.06)	94.08	94.65	94.31	94.19	0.887	0.887
Ferreira et al. (2025) [55]	95.06 (94.37–95.68)	94.72	95.36	94.96	94.84	0.901	0.900
RetinoMamba-AnatomyQuery	97.93 (97.48–98.31)	97.77	98.07	97.87	97.82	0.959	0.959

**Table 5:** External validation performance of the comparative models and the proposed RetinoMamba-AnatomyQuery framework on the independent RFMiD dataset.

Model	Accuracy % (95% CI)	Recall (%)	Specificity (%)	Precision (%)	F1-Score (%)	MCC	Cohen’s κ
Abramovich et al. (2025) [46]	92.30 (91.37–93.19)	91.60	92.80	92.10	92.00	0.846	0.845
Yang et al. (2024) [47]	92.60 (91.69–93.47)	92.10	93.00	92.50	92.40	0.852	0.851
Chaurasia et al. (2025) [48]	91.90 (90.94–92.81)	91.50	92.50	92.00	91.80	0.838	0.837
Saproo et al. (2024) [49]	91.30 (90.31–92.24)	90.80	92.00	91.40	91.10	0.826	0.825
Ferreira et al. (2025) [55] (Vim-Small)	93.70 (92.86–94.49)	94.00	94.50	94.20	94.00	0.874	0.873
RetinoMamba-AnatomyQuery (Vim-Tiny)	96.21 (95.52–96.84)	95.86	96.52	96.13	95.99	0.924	0.924

**Table 6:** Computational complexity comparison of the model.

Model	Backbone/Configuration	Parameters (M)	FLOPs (G)	Inference Time (ms/Image)	Peak GPU Memory (GB)
Abramovich et al. (2025) [46]	CNN-based	25.6	8.9	16.8	2.7
Yang et al. (2024) [47]	ViT-based	86.4	17.6	24.7	4.6
Chaurasia et al. (2025) [48]	Generalised CV architecture	28.3	9.7	18.1	3.0
Saproo et al. (2024) [49]	Transfer-learning CNN	23.8	7.9	15.9	2.6
Ferreira et al. (2025) [55]	Vim-Small reimplementation	26.0	8.8	16.1	2.8
RetinoMamba-AnatomyQuery	Vim-Tiny + EfficientNet-B3 + anatomy-aware modules	23.9	8.3	14.5	2.7

**Table 7:** Grad-CAM++ explainability analysis of RetinoMamba-AnatomyQuery predictions.

Original Image	Ground Truth	Grad-CAM++ Explanation	Prediction	Model Attention Interpretation
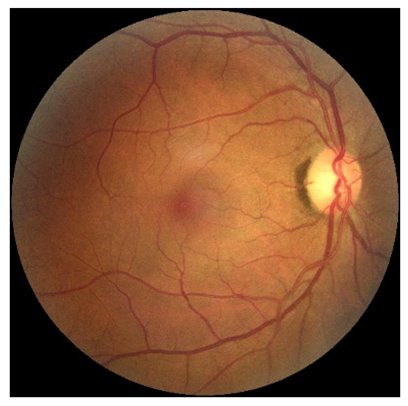	Normal	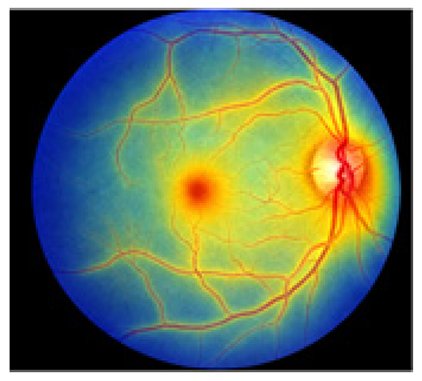	Normal	Activation is concentrated around prominent retinal structures, particularly the optic-disc and central retinal regions, with no distinct activation pattern attributable to an evident pathological region. This supports a structurally informed normal classification.
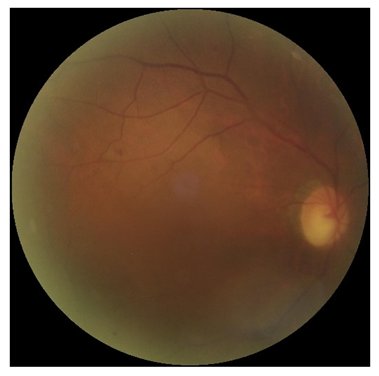	Abnormal	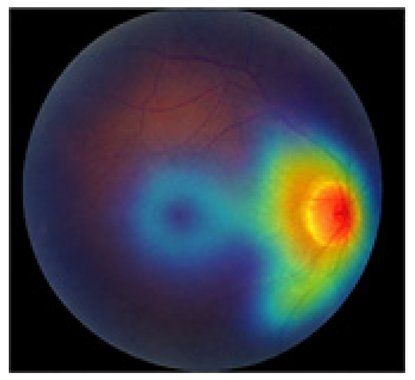	Abnormal	Localized high-intensity activation is observed within the retinal region, contributing strongly to the abnormal prediction, indicating that the decision is primarily influenced by spatially confined retinal features rather than the image background.

## Data Availability

ODIR-2019 Dataset, https://odir2019.grand-challenge.org/, accessed on 15 August 2026; BRSET Dataset, https://physionet.org/content/brazilian-ophthalmological/1.0.2/, accessed on 15 August 2026; RFMiD Dataset, https://www.kaggle.com/datasets/andrewmvd/retinal-disease-classification, accessed on 15 August 2026; and Patient-level Training and Evaluation, https://doi.org/10.5281/zenodo.22787341, accessed on 16 September 2026.
